# Medication Use for Childhood Pneumonia at a Children’s Hospital in Shanghai, China: Analysis of Pattern Mining Algorithms

**DOI:** 10.2196/12577

**Published:** 2019-03-22

**Authors:** Chunlei Tang, Huajun Sun, Yun Xiong, Jiahong Yang, Christopher Vitale, Lu Ruan, Angela Ai, Guangjun Yu, Jing Ma, David Bates

**Affiliations:** 1 Division of General Internal Medicine and Primary Care Brigham and Women’s Hospital Harvard Medical School Boston, MA United States; 2 Children’s Hospital of Shanghai Shanghai Jiaotong University School of Medicine Shanghai China; 3 Shanghai Key Laboratory of Data Science School of Computer Science Fudan University Shanghai China; 4 Shanghai Shenkang Hospital Development Center Shanghai China; 5 Clinical Informatics for the Integrated Health Model Initiative American Medical Association Chicago, IA United States; 6 Department of Population Medicine Harvard Medical School Boston, MA United States

**Keywords:** drug therapy, combination, computer-assisted, pattern recognition, data mining, precision medicine, childhood pneumonia, hospital

## Abstract

**Background:**

Pattern mining utilizes multiple algorithms to explore objective and sometimes unexpected patterns in real-world data. This technique could be applied to electronic medical record data mining; however, it first requires a careful clinical assessment and validation.

**Objective:**

The aim of this study was to examine the use of pattern mining techniques on a large clinical dataset to detect treatment and medication use patterns for childhood pneumonia.

**Methods:**

We applied 3 pattern mining algorithms to 680,138 medication administration records from 30,512 childhood inpatients with diagnosis of pneumonia during a 6-year period at a children’s hospital in China. Patients’ ages ranged from 0 to 17 years, where 37.53% (11,453/30,512) were 0 to 3 months old, 86.55% (26,408/30,512) were under 5 years, 60.37% (18,419/30,512) were male, and 60.10% (18,338/30,512) had a hospital stay of 9 to 15 days. We used the FP-Growth, PrefixSpan, and USpan pattern mining algorithms. The first 2 are more traditional methods of pattern mining and mine a complete set of frequent medication use patterns. PrefixSpan also incorporates an administration sequence. The newer USpan method considers *medication utility*, defined by the dose, frequency, and timing of use of the 652 individual medications in the dataset. Together, these 3 methods identified the top 10 patterns from 6 age groups, forming a total of 180 distinct medication combinations. These medications encompassed the top 40 (73.66%, 500,982/680,138) most frequently used medications. These patterns were then evaluated by subject matter experts to summarize 5 medication use and 2 treatment patterns.

**Results:**

We identified 5 medication use patterns: (1) antiasthmatics and expectorants and corticosteroids, (2) antibiotics and (antiasthmatics or expectorants or corticosteroids), (3) third-generation cephalosporin antibiotics with (or followed by) traditional antibiotics, (4) antibiotics and (medications for enteritis or skin diseases), and (5) (antiasthmatics or expectorants or corticosteroids) and (medications for enteritis or skin diseases). We also identified 2 frequent treatment patterns: (1) 42.89% (291,701/680,138) of specific medication administration records were of intravenous therapy with antibiotics, diluents, and nutritional supplements and (2) 11.53% (78,390/680,138) were of various combinations of inhalation of antiasthmatics, expectorants, or corticosteroids. Fleiss kappa for the subject experts’ evaluation was 0.693, indicating moderate agreement.

**Conclusions:**

Utilizing a pattern mining approach, we summarized 5 medication use patterns and 2 treatment patterns. These warrant further investigation.

## Introduction

Childhood pneumonia remains the single largest cause of death in young children worldwide [1,2]. According to a recent World Health Organization (WHO) report, an estimated 922,000 children under the age of 5 passed away because of pneumonia in 2015 alone, accounting for 16% of all deaths in this age group [2,3]. In China, it is an especially grave concern, driven in part by environmental pollution [3] and abuse of antibiotics [4]. For virtually all high-mortality settings, the WHO’s case management plan uses algorithms as the basis for pneumonia management [5]. Globally, there are many clinical recommendations for treating pediatric pneumonia, but some guidelines are outdated and can be vague [6]. Variation in treatment regimens within the same clinic could indicate poor clinical practice leading to increased recurrence rates that are more likely to have complications [7,8]. Multiple medication therapies are common practice and may provide more effective treatment with a lower concentration of individual components lessening the risk of side effects and toxicity [9,10]. What is missing, however, is the knowledge derived directly from real-world clinical practice.

Electronic medical record (EMR) data represent a rich source of information that includes 2 types of clinical knowledge [8,11,12]. First, it reflects the medical knowledge– and specialty-based clinical practice of a group of doctors within a certain time period. This encompasses medical solutions originating from various clinical standards developed by physicians as a professional group. Second, it represents the clinical experience of an individual practicing clinician and their prescribing style, as well as external factors such as pricing and regulation. Meaningful use of these data can yield information that can guide clinical practice. Challenges of using EMR data, however, have included the large data volume and the substantial variations between different EMR systems.

Data mining techniques have great potential for use in exploring EMR data. Pattern mining is an important subfield of data mining. Its goal is to find all possible salient and persistent patterns in a dataset [13,14], including, but not limited to, direct or indirect associations, trends, periodic patterns, sequential rules, and high-utility patterns of real-life events. Pattern mining is an overloaded term that is used to refer to different technologies in different domains. These techniques have been used in economics, for example, to analyze the stock market and supermarket sales, but have rarely been used in medical research and, specifically, aiming to a variable length of hospital stay is the first time. In one context, it means statistical properties for handling continuous attributes, and in another context, it refers to an ordinal relation, usually based on temporal or spatial precedence that exists among events occurring in the data. Furthermore, the tools and evaluation methods used in each context are different. We aimed to consider the sequential information that may be valuable for identifying recurring features of a dynamic system or predicting future occurrences of certain events. In addition, most of the previous research on discovering medication-using patterns involves text-based methods and is limited to one or two medications [15,16]. In this study, we explored the utility of a pattern mining approach to the entire record of inpatient EMR-based medications administered for childhood pneumonia in a large children’s hospital in China across several years.

## Methods

### Study Population

The Children’s Hospital of Shanghai (CHS), one of the top comprehensive children’s hospitals in China, admits approximately 5000 inpatients annually from different parts of the country and internationally. Formerly known as Underprivileged Children’s Hospital, it is one of the oldest children’s hospitals in Asia.

We extracted 680,138 inpatient medication administration records from 30,512 childhood patients with an initial diagnosis of pneumonia, from January 1, 2010, to December 31, 2015, at the CHS, China. The EMR dataset contains 18,419 males and 12,093 females, among which 60.10% (18,338/30,512) had a hospital stay of 9 to 15 days. If rehospitalized, the patients had 1 record for each hospitalization. Raw data were deidentified. We pulled age and sex, initial diagnosis, and admission and discharge dates, as well as routes and doses of administered medications. According to Chinese legislation, ethical approval from the regional ethical review board is not needed for this type of study of deidentified EMR data. The data used in this study were anonymous. Although ethical approval from the regional ethical review board is not needed for this type of study of deidentified EMRs according to Chinese legislation, we still applied for and received approval from the Institutional Review Board of the CHS.

### Data Preparation

We first merged similar routes of administration for the same medication, and then converted commonly used medications to their shortened reference names as shown in Multimedia Appendix 1. We then removed diluents (or carriers) from the dataset after our expert panel determined that these were not intended to have a therapeutic effect. The final dataset contains 652 individual medications (including traditional Chinese patent medicine [17]), among which the top 40 medications appeared in 73.66% (500,982/680,138) of all the medication administration records (Multimedia Appendix 1).

### Selected Pattern Mining Algorithms: FP-Growth, PrefixSpan, and USpan

Our initial goal was to generate sets of frequently appearing items (ie, frequent item sets) to better understand *medication use patterns*. However, doing so would involve excluding too many temporal data points. An example of this temporal information is the time at which an item (eg, medication) was prescribed by a physician. Thus, while the classic frequency-based framework often leads to many patterns being identified, most of them are not informative enough for further clinical investigation. Recent efforts have been made to incorporate utility into the pattern selection framework, so that high-utility patterns, regardless of frequency, are mined. To obtain more reliable and relevant results, we first developed a unified platform to draw a parallel comparison among all medication use patterns produced by 3 pattern mining algorithms (FP-Growth, PrefixSpan, and USpan). We then asked the panel of subject matter experts to review these results based on their clinical expertise and to make recommendations for meaningful grouping. We also compared these results with what experts found using summary statistics in our previous work [18].

The advantage of progressively using 3 algorithms instead of applying only 1 is twofold: (1) the 3 methods allow for more comprehensive data mining that includes frequency, timing, and utility of dosing of medication administration and (2) it can reduce approach-specific limitations. FP-Growth and PrefixSpan are 2 classic pattern mining algorithms, which were commonly used in business. The earliest and most classic example of their use was examining the cosale of diapers and beer [19]. FP-Growth allows mining of a complete set of frequent patterns by pattern fragment growth without using candidate generations [20]. PrefixSpan offers ordered growth and reduced projected databases over a particular interval [21]. However, the 2 algorithms can only find patterns that appear with high frequency. For example, if there is a medication use pattern of (*a*, *b*) where one of the medications (ie, *a* or *b*) was administered with low frequency, the 2 algorithms would not be able to discover this combination. It is possible that some interesting patterns (eg, a medication prescribed at a low frequency but at a high dose) may be filtered out of the results because of the medication’s low frequency of appearance. To address this problem, we used the more advanced USpan algorithm (first proposed by Yin et al [22]) and proposed a new definition called *medication utility*. The term *utility* originated from economics and considers quantities, profits, and time orders of items simultaneously [23]. The method is unique in its business consideration in dollar value for customers in financial markets. We define *medication utility* using both the dosage and frequency of medication administration over time to obtain more detailed evidence of medication use. USpan reflects a recent effort to incorporate utility into the pattern selection framework, so that high-utility (frequent or infrequent) patterns are mined which address some of the concerns involved in exploratory factor analysis, such as dollar value associated with each pattern. To achieve this, we implemented the USpan algorithm (see Multimedia Appendix 2 with its explanation).

### Application of Pattern Mining Algorithms to the Electronic Medical Record Data

**Figure 1:**
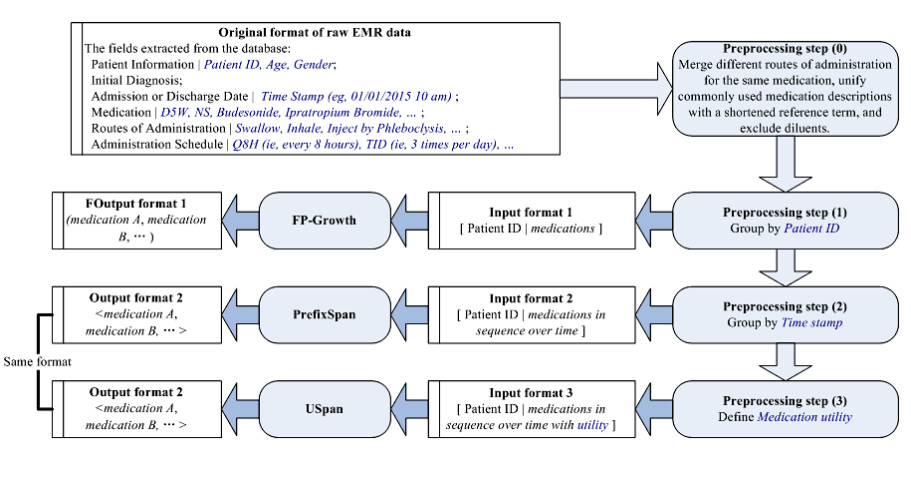
Application of algorithms to the dataset (medication A is a placeholder for a drug name and a indicates the frequency of appearance of medication A). EMR: electronic medical record; D5W: Dextrose 5% in Water; NS: normal saline; ID: identification.

We summarized our approach in Figure 1, including (1) the original format of the raw EMR data, (2) a diagram outlining the 3-step data processing method, and (3) the steps taken by a particular algorithm, and its input and output formats. The thresholds used in the algorithms are as follows: the minimum support of both FP-Growth and PrefixSpan is 0.15, and the minimum utility of USpan is 30,000. We only utilized the top 10 results from our algorithmic outputs.

#### Step 1: Group by Patient Identification

As FP-Growth disregards the quantity of items and time information, we first simplified the raw EMR data to a list of all the administered medications and their corresponding patients. FP-Growth outputs an unordered pattern represented by several medications enclosed in parentheses, for example, (*medication A*, *medication B*, and *medication C*). For example, consider 2 patients, one who used *medication A* on January 1 and *medication B* on January 2, and another who used *medication B* on January 10 and *medication A* on January 21. In this scenario, the output of FP-Growth would be (*medication A* and *medication B*), meaning that *medication A* and *medication B* were administered during the same hospitalization.

#### Step 2: Group by Time Stamp

To make a suitable input for PrefixSpan that includes temporality, we took the results from Step 1 and determined the sequence of medication administration over time. PrefixSpan outputs a temporal order enclosed in a pair of angled brackets. For example, the combination <(*medication A*, *medication B*), *medication C*,...> means that there exists a temporal relation between (*medication A*, *medication B*) and *medication C*. Put another way, *medication A* and *medication B* were administered at the same time, and both were administered before *medication C*. For example, if a patient uses *medication A* for the first day, *medication B* for the first day, *medication D* for the fifth day, *medication A* for the eighth day, and *medication B* for the ninth day, while FP-Growth would output the grouping (*medication A*, *medication B*, *medication D*) without considering the repeat medications, PrefixSpan’s output would be of the form <(*medication A*, *medication B)*, *medication D*, *medication A*, *medication B>*.

#### Step 3: Define Medication Utility

We defined *medication utility* as follows. Let *I={i*_*1*_*, i*_*2*_*,* *...,* *i*_*n*_*}* be the universal set of distinct medications. Each item *i*_*k*_*∈* *I (1≤k≤n)* is associated with a utility value, denoted as *P* (*i*_*k*_), which shows the utility of dosing of each specific medication to treat a given disease. A medication usage is represented as an ordered pair (*i*_*k*_*, q*_*k*_), where *i*_*k*_ *∈* *I* is a medication and *q*_*k*_ is a positive number representing the quantity of *I­*, which shows how much of this medication is taken (ie, dose). We thus define *medication utility* as *U(i*_*k*_*, q*_*k*_*)=P(i*_*k*_*)* *×* *q*_*k*_ representing a single medication’s dosage during one patient’s hospitalization.

Since USpan takes both the time sequence and medication utility of each item into account, every administration record that occurred at the same time will be enclosed in a pair of parentheses, such as (*medication A*, *a*), in which *medication A* is a single drug and *a* is the frequency of *medication A*’s appearance. Each medication is attached to its utility, and each medication-utility pair is separated by time, indicated by a comma. USpan results are in the same output format as PrefixSpan.

### Experts’ Reviewing of the Results

To determine which machine-identified patterns most accurately reflect real-world clinical practice [24], we invited a panel of subject matter experts including 3 physicians (MD), 3 pharmacists, and 2 researchers (1 DS and 1 MPH). The panelists were invited to review, adjusting process inputs and outputs if needed, and offer opinions on the clinical validity of the patterns obtained from the 3 algorithms. We then conducted a thorough literature review (by putting the drug names seen in a specific pattern into Google’s search engine to find related papers) to further analyze the combinations that our panel considered clinically interesting. Our experts then conducted a final review of the results of the literature search to further validate the medication use patterns. We calculated Fleiss kappa to measure interrater reliability for the experts shown in Multimedia Appendix 3.

## Results

### Study Population

The majority (86.55%, 26,408/30,512) of the study population (Table 1) was under 5 years of age. Among the under-5 population, 66.55% (20,305/30,512) were under 2 years of age and 37.53% (11,453/30,512) were newborns. There were more male (60.37%, 18,419/30,512) than female patients.

### Simple Exploration of Frequency of Use of the Raw Electronic Medical Record Data

To better understand the raw EMR data, we first examined the top 40 most frequently prescribed medications across age groups and found that 2 diluents (D5W, Dextrose 5% in Water and NS, Normal Saline) were ranked the first- and second-most frequent (Multimedia Appendix 4). After removing all diluents from the dataset, some nutritional supplements, such as fat emulsion and vitamins, were ranked within top 10 medications for each of the 6 age groups (Multimedia Appendix 5). Besides diluents and nutritional supplements, antibiotics were also among the top 10 most frequently prescribed in each age group. These medications were present in 42.89% (291,701/680,138) of all specific medication administration records and they were all administered through intravenous (IV) therapy. Thus, *treatment pattern 1* is the combination of antibiotics, diluents, and nutritional supplements via IV therapy.

**Figure 2:**
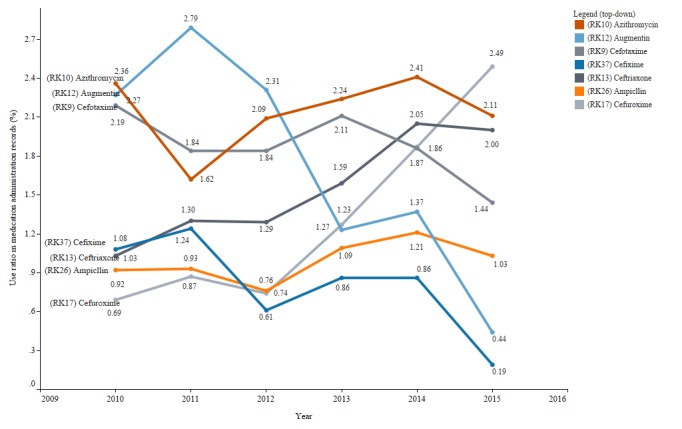
The historic proportion of antibiotics administered according to the calendar year.

**Figure 3:**
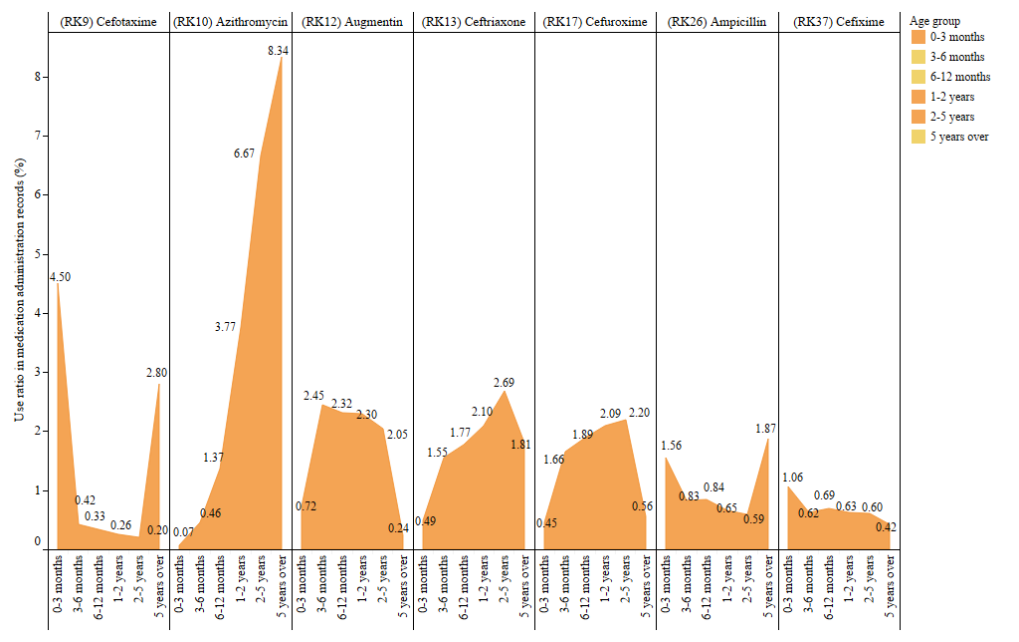
The historic proportion of antibiotics administered according to patient age group.

Due to the high frequency of antibiotic use, we then used statistics on individual medications to examine the frequency of use of the 7 commonly administered antibiotic monotherapies over the study period (2010 to 2015) and found a dramatically increased use of ceftriaxone (a third-generation cephalosporin antibiotic) and cefuroxime (a second-generation cephalosporin) over this period of time (Figure 2). Meanwhile, the use of a more traditional first-generation antibiotic—augmentin—had dramatically declined. In addition, we found that the administration of certain types of antibiotics varied with patients’ age (Figure 3). For instance, cefotaxime (a third-generation cephalosporin) was more commonly used in newborns under 3 months of age, but azithromycin was rarely administered in that age group. This observation might reflect the results of some published studies which reported an increased risk of cardiovascular death associated with the use of azithromycin, specifically, in infants [25,26].

**Table 1:** Study population demographics.

Characteristics		Year					
		2010	2011	2012	2013	2014	2015
Total population (N)		4216	4062	3935	5080	6198	7021
**Sex, n (%)**							
	Male	2602 (61.72)	2447 (60.24)	2411 (61.27)	3030 (59.65)	3718 (59.99)	4211 (59.98)
	Female	1614 (38.28)	1615 (39.76)	1524 (38.73)	2050 (40.35)	2480 (40.01)	2810 (40.02)
**Age groups^a^**, **n (%)**							
	0-3 months	1678 (39.80)	1617 (39.81)	1609 (40.89)	2075 (40.85)	2297 (37.06)	2177 (31.01)
	3-6 months	356 (8.44)	413 (10.17)	360 (9.15)	351 (6.91)	410 (6.62)	549 (7.82)
	6-12 months	441 (10.46)	491 (12.09)	386 (9.81)	482 (9.49)	580 (9.36)	716 (10.20)
	1-2 years	426 (10.10)	421 (10.36)	421 (10.70)	504 (9.92)	723 (11.67)	822 (11.71)
	2-5 years	781 (18.52)	725 (17.85)	711 (18.07)	902 (17.76)	1279 (20.64)	1705 (24.28)
	>5 years	534 (12.67)	395 (9.72)	448 (11.39)	766 (15.08)	909 (14.67)	1052 (14.98)

^a^Age indicates the age at admission and was calculated as the difference between *Admission Date* and *Birthday*.

### Using Pattern Mining to Explore the Electronic Medical Record Dataset

We utilized the 3 algorithms to produce the top 10 medication use patterns from 6 age groups spanning from 0 to 17 years, resulting in 180 distinct medication combinations (Multimedia Appendix 1). On the basis of these results, our expert panel summarized 5 clinically interesting medication use patterns as shown in Table 2. Checkmarks indicate that the pattern appeared in that age group. *P* *attern 1* is antiasthmatics (albuterol) and expectorants (ipratropium bromide) and corticosteroids (budesonide). *Pattern 2* is antibiotics and (antiasthmatics or expectorants or corticosteroids). *Pattern 3* is the only use of third-generation antibiotics or the use of these medications followed by traditional antibiotics. *Pattern 4* is antibiotics and medications for enteritis (probiotics: bifid triple viable, smectite, and clostridium butyricum) or medication for skin diseases (antiseptics: zinc oxide and drapolene). Finally, *Pattern 5* is (antiasthmatics or expectorants or corticosteroids) and (medications for enteritis or skin diseases). Detailed descriptions of the patterns for 3 major groups are provided in the following sections.

### Inhaled Medications

Medication use patterns 1 and 2 were both revealed by USpan, because it takes into account a high medication utility for administered medications (Multimedia Appendix 5). For pattern 1, we found that all 3 medications reflect nonsequential medication administrations, indicating that they were administered concurrently instead of the more traditional sequential application of each medication. Our expert panel confirmed that this medication pattern could shorten medication administration durations, for example by combining and administering multiple inhaled medications simultaneously via nebulization. This approach was also confirmed by the chemical stability of such a mixture [27,28]. Further investigation of the route of administration of pattern 1 medication use indicated that inhalation therapy accounted for 11.53% (78390/680138) of the medication administration records, which we considered as *Treatment pattern 2*. Medication pattern 2 was these inhaled medications plus antibiotics indicating the management pattern of difficult-to-treat infections in patients with pneumonia.

### Antibiotics

FP-Growth and PrefixSpan, the 2 classical algorithms, showed a similar pattern 3 (Table 2), that is, the only use of third-generation antibiotics or the use of these medications with (or followed by) traditional antibiotics. FP-Growth resulted in 2 combinations. One is the combination of 2 third-generation cephalosporins (cefotaxime and cefixime). The other is the combination of a third-generation cephalosporin (ceftriaxone) with a traditional first-generation antibiotic (azithromycin). Interestingly, PrefixSpan showed not only the same 2 medication combinations but also similar timings: the average time gap between the 2 medications was 7 and 5 days, respectively.

### Medications for Enteritis or Skin Diseases

Pattern**s** 4 and 5 showed a correlation between pneumonia-specific medications and medications for 2 other conditions: enteritis and skin diseases. For example, pattern 4 demonstrated a third-generation antibiotic (cefotaxime) and a medication for skin rashes (zinc oxide) were followed by a probiotic (bifid triple viable) that appears only in the *0 to 3 months* age group. Pattern 5 showed that the 3 inhalation medications (albuterol, ipratropium bromide, and budesonide) were followed by probiotics without using antibiotics that mainly appeared in the *3 to 6 months* age group. Due to the fact that PrefixSpan only determines the order of different medications without considering a time window, we further examined the time interval between medications for pneumonia following medication for enteritis or skin diseases (Multimedia Appendix 4). The findings revealed that the average time intervals were similar (4 vs 6 days) in the abovementioned 2 examples for patterns 4 and 5.

**Table 2:** A select list of clinically interesting results produced by 3 pattern mining algorithms.

#	Medication use patterns	FP-Growth	PrefixSpan	USpan	Age groups					
					0-3 m	3-6 m	6-12 m	1-2 y	2-5 y	>5 y
1	anti-asthmatics AND expectorants AND corticosteroids	(Albuterol, Ipratropium Bromide, Budesonide)	(Albuterol, Ipratropium Bromide, Budesonide)	(Albuterol, Ipratropium Bromide, Budesonide), (Pholcodine)	✓^a^	✓	✓	✓	✓	✓
2	antibiotics AND (anti-asthmatics OR expectorants OR corticosteroid)	—^b^	—	(Albuterol, Ipratropium Bromide, Budesonide), (Azithromycin)			✓	✓	✓	✓
		—	—	(Albuterol, Ipratropium Bromide, Augmentin, Budesonide), (Azithromycin)						✓
3	third-generation cephalosporin antibiotics with (or followed by) traditional antibiotics	(Cefotaxime, Cefixime)	(Cefotaxime), (Cefixime)	—	✓					
		(Ceftriaxone, Azithromycin)	(Ceftriaxone), (Azithromycin)	—						✓
4	anti-biotics AND (medications for enteritis OR skin diseases)	(Bifid Triple Viable, Cefotaxime, Zinc Oxide)	(Cefotaxime, Zinc Oxide), (Bifid Triple Viable)	—	✓					
		(Bifid Triple Viable, Cefotaxime, Drapolene)	(Bifid Triple Viable, Cefotaxime)	—	✓					
		(Cefotaxime, Zinc Oxide, Drapolene)	(Cefotaxime, Zinc Oxide, Drapolene)	—	✓					
		(Smecitite, Bifid Triple Viable, Cefotaxime)	—	—	✓					
5	(antiasthmatics OR expectorants OR corticosteroids) AND (medications for enteritis OR skin diseases)	(Bifid Triple Viable, Albuterol, Ipratropium Bromide, Budesonide)	(Albuterol, Ipratropium Bromide, Budesonide), (Bifid Triple Viable)	—		✓				

^a^✓ indicates that the pattern appeared within the specified age groups.

^b^No result from the specific algorithm.

## Discussion

### Principal Findings

There is a considerable demand for appropriate and proven approaches to expanding the use of medication analytics. By simply checking the frequency of use of medications in the raw EMR data, we found that IV administration is the most common administration route (treatment pattern 1) for treating inpatient childhood pneumonia in this hospital. This finding is in line with concerns about heavy use of IV therapy in China reported by the WHO [29]. Even then, statistical analysis is limited in its ability to discover relationships among medications, and it typically only produces a list of monotherapies with frequency rankings [30]. Indeed, one could argue that from the perspective of treating a given disease, a medication administered with higher frequency implies higher efficacy. However, our results illustrate that this is not always the case.

By utilizing 3 pattern mining algorithms (FP-Growth, PrefixSpan, and USpan), which are commonly used for business applications, we not only found 5 medication use patterns but also identified an additional treatment pattern (treatment pattern 2), that is, medication administration via inhalation route.

There were differing opinions within our expert panel as to what pattern 4 (antibiotics and medications for enteritis or skin diseases) indicates. One possible explanation, offered by 4 US experts, is that because of the immaturity of infant organs, damage to one organ or organ system could cause reactions in other organs [31]. Furthermore, from a pharmaceutical perspective, broad-spectrum antibiotics (eg, cefotaxime) cause diarrhea by irradiating normal gastrointestinal (GI) flora [32], and physicians can treat this type of diarrhea in the pediatric population with bifid triple viable, a probiotic used to re-establish normal GI flora. Additionally, diarrhea can cause diaper rash that can be treated with zinc oxide. Chinese experts believed that these findings support an underlying internal relationship between the lungs and large intestine (or the lungs and skin), which is consistent with established theories of traditional Chinese medicine (TCM) [33]. TCM believes that the human body is an organic whole, that there is a relationship between the lung and large intestine, and that the lungs govern the skin and hair [34]. Despite this rationale, our expert panel generally agreed that pattern 5 was unexpected (ie, the use of medications for enteritis or skin diseases following inhalation treatment, without concurrent use of antibiotics). The overall Fleiss kappa for these 7 patterns was 0.693 (Table 2) indicating moderate agreement [35]. Our findings neither contradict nor confirm these hypothesized relationships as our data target treatment protocols which cannot, on their own, speak to the relationship between organ systems. However, according to a recent study on innate lymphoid cells, lung inflammation might originate in the gut [36], and there exists a link between intestinal microbiota and lung diseases (eg, asthma) [37].

Among the strengths of this study is its novel application of the 3 pattern mining algorithms to medical data. We determined 5 medication use patterns that can succinctly reflect prescriber style among complex real-world hospital EMR use. This prescriber style reflects decision factors beyond efficacy (eg, availability and cost of a medication, frequency of administration in a busy setting, and hospital formulary). Although patients with an initial EMR diagnosis of *pneumonia* could include all varieties of pneumonia to which various treatment protocols may apply, most of our results correlated with our literature searches regarding the medications’ use. For example, a combination of 2 antibiotics produced by FP-Growth (cefuroxime and azithromycin) was evaluated by Vergis et al [38], who found no increased risk of mortality associated with prescribing the 2 antibiotics continuously or simultaneously. Another example is by Rubio et al [39], which evaluated the sequential combination of cefotaxime and cefixime, finding that prescribing them within 2 to 3 days of each other may result in shorter hospital stays, a pattern that was also found by PrefixSpan. Results that have not been mentioned in the current literature (eg, pattern 3) warrant further investigation.

One limitation of this study is that we focused on one source of data from a single pediatric hospital in Shanghai, China. Although other pediatric hospitals in China might not have unique medical practices, this Shanghai-based pediatric hospital has been the leading medical institution with a strong link to the international pediatric community; it contains up-to-date technology, and its clinicians undergo continuous medical training. Our findings provide firsthand insight into understanding Chinese pediatricians’ experiences in the treatment of pneumonia. Although our results may not be generalizable across all demographics, especially in remote areas of China, it is likely that most medication use patterns are universal in the more populated areas of China.

Our work is the first step toward better synthesis of current practice and establishing more realistic treatment protocols for childhood pneumonia. Moving forward, we plan to expand this study within and across childhood pneumonia groups from multiple health care organizations. We also envision that a knowledge base of medication use patterns will serve as an informative guide to researchers and clinicians. Our discovered treatment patterns have the potential for inclusion in treatment protocols. We hope to further integrate clinical and nonclinical information into our algorithms to help determine cost and efficacy of treatments, as well as readmission, mortality, and morbidity rates.

### Conclusions

We used a pattern mining approach to automatically acquire knowledge of prior medication treatment combinations for childhood pneumonia. An expert panel summarized 5 medication use patterns. These, together with 2 identified treatment patterns that also targeted skin disease and enteritis, may warrant further investigation. Additionally, our findings suggest the following starting points for further discussion: (1) a comparison of IV therapy before and after the publication of China’s new deal on the rational use of medicines, the *2013 Principle of Rational Use of Medicines* [40], (2) validation and comparison of efficacy of various medication use patterns, and (3) a potential relationship between the lungs and skin.

## Appendix

Multimedia Appendix 1 The implementation of the USpan algorithm.

Multimedia Appendix 2 Related commonly administered medications with their shortened reference names.

Multimedia Appendix 3 Values for Fleiss kappa (N=7, n=8, k=2) as substantial agreement.

Multimedia Appendix 4 Appearance of “medications for enteritis or skin diseases” in pneumonia treatment course within patients’ medication administration records.

Multimedia Appendix 5 The top 10 results distinguished by age groups.

## Abbreviations

CHS: Children’s Hospital of Shanghai
EMR: electronic medical record
GI: gastrointestinal
IV: intravenous
TCM: traditional Chinese medicine
WHO: World Health Organization
