# Supplementary material for: Medication Use for Childhood Pneumonia at a Children’s Hospital in Shanghai, China: Analysis of Pattern Mining Algorithms
Source: JMIR Med Inform. 2019 Mar 22;7(1):e12577. doi: 10.2196/12577 (PMC6450478; doi:10.2196/12577)
Supplement: Multimedia Appendix 1 [file medinform_v7i1e12577_app1.pdf]

Multimedia Appendix 1. The Implementation of the USpan algorithm

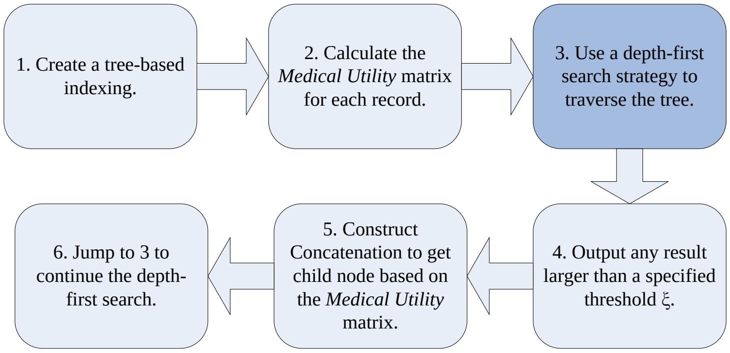

We implemented the USpan algorithm according to the following algorithmic process, then modified it to add support for medicine constraints. USpan uses a tree-based mining approach to store the utility information of items in quantitative sequences, where each node in the tree is a medication administration record along with its utility. Initially, the tree only contains nodes with one medication. Next, USpan uses a depth-first search strategy to traverse the LQS-Tree. Each node's value is compared to a specified threshold ξ; if the node's value is lower than ξ, then it returns to its parent nodes. In this study, we used the top k high utility sequential patterns instead of using the threshold ξ. Both width pruning and depth pruning are employed using the medication utility matrix.
