# Supplementary material for: Medication Use for Childhood Pneumonia at a Children’s Hospital in Shanghai, China: Analysis of Pattern Mining Algorithms
Source: JMIR Med Inform. 2019 Mar 22;7(1):e12577. doi: 10.2196/12577 (PMC6450478; doi:10.2196/12577)
Supplement: Multimedia Appendix 2 [file medinform_v7i1e12577_app2.pdf]

Multimedia Appendix 2. Related Commonly Administered Medications with Their Shortened Reference Names

EMR Rank[a]	Records (%)[b]	Medication Reference Name[c]	Medication Type	Medication Description
1	55,288 (8.13)	D5W	diluents	100 mL of 5% Dextrose Injection
2	50,863 (7.48)	NS	diluents	100 mL of 0.9% Sodium Chloride Injection
3	30,730 (4.52)	Budesonide	corticosteroids	Budesonide Inhalation Suspension<br>*It works directly in the lungs to make breathing easier by reducing the irritation and swelling of the airways.*
4	27,532 (4.05)	Ipratropium Bromide	expectorants	Ipratropium Bromide Inhalation Solution<br>*It is used to control and prevent symptoms (wheezing and shortness of breath) caused by ongoing lung disease (chronic obstructive pulmonary disease-COPD which includes bronchitis and emphysema).*
5	23,711 (3.49)	NaCl	nutritional supplements	10% Sodium Chloride Concentrated Solution for Injection
6	23,227 (3.42)	KCl	nutritional supplements	10% Potassium Chloride injection
7	20,128 (2.96)	Albuterol	antiasthmatics	Albuterol Sulfate Inhalation Solution<br>*A class of medications known as bronchodilators.*
8	15,526 (2.28)	Bifid Triple Viable*	probiotics	Bacillus Acidophilus, Bifidobacterium Bifidum, Fecal Streptococci Powder<br>*It is used for many conditions affecting the intestines, including preventing diarrhea in infants and children.*
9	13,027 (1.92)	Cefotaxime	antibiotics	Cefotaxime Sodium for Injection
10	12,675 (1.86)	Azithromycin	antibiotics	Azithromycin Suspension, Injection, and Tablets
11	11,309 (1.66)	SWFI	diluents	Sterile Water for Injection
12	11,155 (1.64)	Augmentin	antibiotics	Amoxicillin Sodium/Potassium Clavulanate (Injections)
13	11,023 (1.62)	Ceftriaxone	antibiotics	Ceftriaxone Sodium for Injection
14	10,679 (1.57)	Chymotrypsin	antiasthmatics	Chymotrypsin for Injection<br>*It is an enzyme that helps loosen phlegm in asthma, bronchitis, lung diseases, and sinus infections.*
15	10,588 (1.56)	Ibuprofen	anti-inflammatory agents	Ibuprofen Oral Suspension and Drops<br>*It is a nonsteroidal anti-inflammatory medication (NSAID) that possesses anti-inflammatory, analgesic and antipyretic activity.*
16	10,528 (1.55)	Ambroxol	expectorants	Ambroxol Hydrochloride Injection<br>*It is a clinically proven systemically active mucolytic agent. The breakdown of acid mucopolysaccharide fibers makes the sputum thinner and less viscous and therefore more easily rEMRved by coughing.*

EMR Rank[a]	Records (%)[b]	Medication Reference Name[c]	Medication Type	Medication Description
**17**	10,116 (1.49)	Cefuroxime	antibiotics	Cefuroxime Sodium for Injection
**18**	9,957 (1.46)	Dexamethasone	corticosteroids	Dexamethasone Sodium Phosphate Injection
**19**	8,795 (1.29)	25% Glucose Injection	nutritional supplements	100 mL of 25% Glucose Injection
**20**	8,762 (1.29)	Smecitite*	probiotics	Smectite/Montmorillonite Powder *It may be helpful in the treatment of irritable bowel syndrome.*
**21**	8,561 (1.26)	Amino Acid	nutritional supplements	Pediatric Compound Amino Acid injection (19AA-I)
**22**	8,312 (1.22)	Fat Emulsion	nutritional supplements	Fat Emulsion Injection (C14-24)
**23**	7,585 (1.12)	Water-Soluble Vitamins	nutritional supplements	Water-soluble Vitamin for Injection
**24**	7,169 (1.05)	Zinc Oxide†	antiseptics	Zinc Oxide Ointment *It can also be used to treat minor skin irritations (eg, cuts, burns, and scrapes, poison ivy).*
**25**	6,995 (1.03)	Fat-Soluble Vitamins	nutritional supplements	Fat-soluble Vitamin Injection (II)
**26**	6,976 (1.03)	Ampicillin	antibiotics	Ampicillin Sodium/Sulbactam Sodium for Injection
**27**	6,885 (1.01)	Meptin Syrup	antiasthmatics	Procaterol Hydrochloride Oral Solution *Relief of dyspnea and other symptoms caused by respiratory obstructive disturbance in the following diseases: bronchial asthma, chronic bronchitis, and pulmonary emphysema.*
**28**	6,732 (0.99)	Secorine Syrup	antihistamines	Guaifenesin, Methylephedrine and Chlorpheniramine Syrup *It is an antihistamine.*
**29**	6,477 (0.95)	Alanyl Glutamine	nutritional supplements	Alanyl Glutamine Injection
**30**	6,443 (0.95)	Pholcodine	antiasthmatics	Compound Pholcodine Oral Solution *It is a medication which is an opioid cough suppressant (antitussive).*
**31**	6,251 (0.92)	10% Glucose Injection	nutritional supplements	100 mL of 10% Glucose Injection
**32**	6,211 (0.91)	Solu-Medrort	corticosteroids	SOL-MELCORT for injection (Methylprednisolone Sodium Succinate)
**33**	6,180 (0.91)	Chloral Hydrate	tranquilizers	Chloral Hydrate Oral Solution *In children aged 2-11 years treatment should be as an adjunct to behavioural therapy and sleep hygiene management, and usually for duration of less than 2 weeks.*

**EMR Rank**[a]	**Records (%)**[b]	**Medication Reference Name**[c]	**Medication Type**	**Medication Description**
**34**	5,779 (0.85)	Midazolam	tranquilizers	Midazolam Injection<br>*It used for children offer anxiolysis, sedation and amnesia.*
**35**	5,652 (0.83)	Drapolene†	antiseptics	Benzalkonium Chloride and Cetrimonium Bromide Cream<br>*It is a topical antiseptic and works by preventing infection.*
**36**	5,588 (0.82)	Spasmo-Mucosolvan	expectorants	Ambroxol Hydrochloride and Clenbuterol Hydrochloride Oral Solution<br>*It is a clinically proven systemically active mucolytic agent.*
**37**	5,445 (0.80)	Cefixime	antibiotics	Cefixime Granules
**38**	4,738 (0.70)	Solu Cortef	corticosteroids	Hydrocortisone Sodium Succinate for Injection
**39**	3,910 (0.57)	Vitamin K1	nutritional supplements	Vitamin K1 Injection
**40**	3,474 (0.51)	Clostridium Butyricum*	probiotics	Clostridium Butyricum Powder<br>*It may effect on the restoration of the intestinal microbiota.*

[a] Rank was determined by how frequently the medication appeared within our timeframe where 1 is the most frequent.

[b] Records refer to a specific medication administration records among a total of 680,138 records.

[c] Enteritis medications are indicated with an asterisk, and skin medications are indicated with obelisk.
