# Supplementary material for: Medication Use for Childhood Pneumonia at a Children’s Hospital in Shanghai, China: Analysis of Pattern Mining Algorithms
Source: JMIR Med Inform. 2019 Mar 22;7(1):e12577. doi: 10.2196/12577 (PMC6450478; doi:10.2196/12577)
Supplement: Multimedia Appendix 3 [file medinform_v7i1e12577_app3.pdf]

Multimedia Appendix 3. Values for Fleiss'Kappa (N=7, n=8, k=2) as Substantial Agreement.

n_{ij}	Expected	Unexpected	P_i
**Medication Pattern 1**	8	0	1.000
**Medication Pattern 2**	7	1	0.750
**Medication Pattern 3**	8	0	1.000
**Medication Pattern 4**	8	0	1.000
**Medication Pattern 5**	0	8	1.000
**Treatment Pattern 1**	8	0	1.000
**Treatment Pattern 2**	6	2	0.571
**Total**	45	11	6.321
P_j	0.804	0.196	

Note that we calculated Fleiss'kappa to measure inter-rater reliability for five medication patterns and two treatment patterns where the 8 experts reviewed every one. Over the whole table,

$$\overline{P} = \frac{1}{(7)}(6.321) = 0.903$$

$$\overline{P}_{\ell} = 0.804^{2} + 0.196^{2} = 0.684$$

$$\kappa = \frac{0.903 - 0.684}{1 - 0.684} = 0.693$$
