# Supplementary material for: Medication Use for Childhood Pneumonia at a Children’s Hospital in Shanghai, China: Analysis of Pattern Mining Algorithms
Source: JMIR Med Inform. 2019 Mar 22;7(1):e12577. doi: 10.2196/12577 (PMC6450478; doi:10.2196/12577)
Supplement: Multimedia Appendix 4 [file medinform_v7i1e12577_app4.pdf]

Multimedia Appendix 4. Appearance of "Medications for Enteritis OR Skin Diseases" in Pneumonia Treatment Course within Patients' Medication Administration Records.

Pattern	Age groups	Medications for enteritis			Medications for skin diseases		
		# Patients Prescribed	Percentage of All Administered Medications (%)	Duration Post-Admission (days)	# Patients Prescribed	Percentage of All Administered Aedications (%)	Duration Post-Admission (days)
Expected pattern 4 antibiotics AND (medications for enteritis OR skin diseases)	0 to 3 months	3,613	31.5%	5	100	0.9%	5
	3 to 6 months	577	20.8%	4	87	3.1%	6
	6 to 12 months	718	21.9%	4	120	3.7%	6
	1 to 2 years	567	16.5%	4	53	1.5%	4
	2 to 5 years	715	11.6%	8	35	0.6%	4
	5 years and over	320	7.8%	11	15	0.4%	12
Unexpected pattern 5 (anti-asthmatics OR expectorants OR corticosteroids) AND (medications for enteritis OR skin diseases)	0 to 3 months	2,496	21.8%	5	116	1.0%	4
	3 to 6 months	665	24.0%	4	111	4.0%	6
	6 to 12 months	726	22.1%	4	130	4.0%	6
	1 to 2 years	509	14.8%	5	56	1.6%	4
	2 to 5 years	595	9.6%	6	32	0.5%	4
	5 years and over	258	6.2%	8	11	0.3%	11
