# Supplementary material for: Medication Use for Childhood Pneumonia at a Children’s Hospital in Shanghai, China: Analysis of Pattern Mining Algorithms
Source: JMIR Med Inform. 2019 Mar 22;7(1):e12577. doi: 10.2196/12577 (PMC6450478; doi:10.2196/12577)
Supplement: Multimedia Appendix 5 [file medinform_v7i1e12577_app5.pdf]

Multimedia Appendix 5. The Top 10 Results Distinguished by Age Groups

Age Group	Top 10	Summary Statistics	FP-Growth	PrefixSpan	USpan
**Under 2 years**					
**0 to 3 months**	1	Cefotaxime	(Bifid Triple Viable, Cefotaxime, Zinc Oxide)	(Albuterol, Ipratropium Bromide, Budesonide)	(Albuterol, Ipratropium Bromide, Budesonide)
	2	Bifid Triple Viable	(Albuterol, Ipratropium Bromide, Budesonide)	<(Bifid Triple Viable), (Bifid Triple Viable)>	(Ipratropium Bromide, Budesonide)
	3	Budesonide	(Cefotaxime, Zinc Oxide, Drapolene)	(Cefotaxime, Zinc Oxide, Drapolene)	(Cefotaxime)
	4	Fat Emulsion	(Bifid Triple Viable, Budesonide)	(Ampicillin)	(Albuterol, Budesonide)
	5	Amino Acid	(Bifid Triple Viable, Cefotaxime, Chymotrypsin)	<(Cefotaxime, Zinc Oxide), (Bifid Triple Viable)>	(Albuterol, Ipratropium Bromide)
	6	Zinc Oxide	(Cefotaxime, Cefixime)	<(Cefotaxime), (Cefixime)>	(Albuterol, Ipratropium Bromide, Budesonide, Augmentin)
	7	Water-soluble Vitamins	(Smecitite, Bifid Triple Viable, Cefotaxime)	(Bifid Triple Viable, Cefotaxime)	(Albuterol, Budesonide, Augmentin)
	8	Fat-soluble Vitamins	(Vitamin K1)	(Clostridium Butyricum)	(Ipratropium Bromide, Budesonide, Augmentin)
	9	Alanyl Glutamine	(Dexamethasone, Cefotaxime, Chymotrypsin, Zinc Oxide)	(Cefotaxime, Budesonide)	(Ipratropium Bromide, Albuterol, Augmentin)
	10	Ipratropium Bromide	(Bifid Triple Viable, Cefotaxime, Drapolene)	(Chymotrypsin, Cefotaxime, Zinc Oxide)	<(Ipratropium Bromide, Albuterol, Budesonide), (Ipratropium Bromide, Albuterol, Budesonide)>
**3 to 6 months**	1	Budesonide	(Albuterol, Ipratropium Bromide, Augmentin, Budesonide)	(Ceftriaxone)	<(Albuterol, Ipratropium Bromide, Budesonide), (Albuterol, Ipratropium Bromide, Budesonide)>
	2	Ipratropium Bromide	(Bifid Triple Viable, Albuterol, Ipratropium Bromide, Budesonide)	(Albuterol, Ipratropium Bromide, Budesonide)	<(Albuterol, Ipratropium Bromide, Budesonide), (Ipratropium Bromide, Budesonide)>
	3	Albuterol	(Dexamethasone, Chymotrypsin)	(Ambroxol)	<(Albuterol, Ipratropium Bromide, Budesonide), (Albuterol, Ipratropium Bromide)>
	4	Bifid Triple Viable	(Albuterol, Ipratropium Bromide, Budesonide, Meptin Syrup)	(Chymotrypsin, Dexamethasone)	<(Albuterol, Ipratropium Bromide, Budesonide), (Albuterol, Budesonide)>

	**5**	Augmentin	(Cefuroxime, Ipratropium Bromide, Budesonide)	<(Albuterol, Ipratropium Bromide, Budesonide,), (Albuterol, Ipratropium Bromide, Budesonide)>	<(Ipratropium Bromide, Budesonide), (Albuterol, Ipratropium Bromide, Budesonide)>
	**6**	Cefuroxime	(Ceftriaxone, Ipratropium Bromide, Budesonide)	(Spasmo-Mucosolvan)	(Albuterol, Ipratropium Bromide, Augmentin, Budesonide)
	**7**	Midazolam	(Chloral Hydrate)	<(Albuterol, Ipratropium Bromide, Budesonide), (Bifid Triple Viable)>	<(Albuterol, Ipratropium Bromide, Budesonide), (Ipratropium Bromide)>
	**8**	Ceftriaxone	(Ipratropium Bromide, Budesonide, Ambroxol)	(Chloral Hydrate)	<(Ipratropium Bromide, Budesonide), (Budesonide)>
	**9**	Smecitite	(Solu Cortef, Albuterol, Ipratropium Bromide, Budesonide)	(Secodrine Syrup)	<(Albuterol, Ipratropium Bromide), (Albuterol, Ipratropium Bromide, Budesonide)>
	**10**	Solu Cortef	(Ipratropium Bromide, Budesonide, Spasmo-Mucosolvan)	(Budesonide, Meptin Syrup, Ipratropium Bromide)	<(Albuterol, Budesonide), (Albuterol, Ipratropium Bromide, Budesonide)>
	**1**	Budesonide	(Bifid Triple Viable, Albuterol, Ipratropium Bromide, Budesonide)	(Ceftriaxone)	<(Albuterol, Ipratropium Bromide, Budesonide), (Albuterol, Ipratropium Bromide, Budesonide)>
	**2**	Ipratropium Bromide	(Albuterol, Ipratropium Bromide, Budesonide, Meptin Syrup)	(Smecitite)	<(Albuterol, Ipratropium Bromide, Budesonide), (Ipratropium Bromide, Budesonide)>
	**3**	Albuterol	(Albuterol, Ipratropium Bromide, Augmentin, Budesonide)	(Ambroxol)	<(Ipratropium Bromide, Budesonide), (Albuterol, Ipratropium Bromide, Budesonide)>
**6 to 12 months**	**4**	Ibuprofen	(Ceftriaxone, Ipratropium Bromide, Budesonide)	(Albuterol, Ipratropium Bromide, Augmentin, Budesonide)	<(Albuterol, Ipratropium Bromide, Budesonide), (Albuterol, Budesonide)>
	**5**	Bifid Triple Viable	(Cefuroxime, Ipratropium Bromide, Budesonide)	(Ipratropium Bromide, Budesonide, Meptin Syrup)	<(Albuterol, Ipratropium Bromide, Budesonide), (Albuterol, Ipratropium Bromide)>
	**6**	Augmentin	(Chloral Hydrate)	(Chloral Hydrate)	(Albuterol, Ipratropium Bromide, Augmentin, Budesonide)
	**7**	Cefuroxime	(Ipratropium Bromide, Budesonide, Ambroxol)	(Azithromycin)	<(Albuterol, Ipratropium Bromide, Budesonide), (Ipratropium Bromide)>
	**8**	Ceftriaxone	(Solu Cortef, Ipratropium Bromide, Budesonide)	(Spasmo-Mucosolvan)	<(Albuterol, Budesonide), (Albuterol, Ipratropium Bromide, Budesonide)>

	**9**	Meptin Syrup	(Dexamethasone, Chymotrypsin)	<(Albuterol, Ipratropium Bromide, Budesonide), (Albuterol, Ipratropium Bromide, Budesonide)>	<(Albuterol, Ipratropium Bromide), (Albuterol, Ipratropium Bromide, Budesonide)>
	**10**	Smecitite	(Ibuprofen)	(Solu Cortef)	<(Albuterol, Budesonide), (Ipratropium Bromide, Budesonide)>
**12 to 24 months**	**1**	Budesonide	(Albuterol, Ipratropium Bromide, Azithromycin, Budesonide)	(Bifid Triple Viable)	<(Albuterol, Ipratropium Bromide, Budesonide), (Azithromycin)>
	**2**	Ipratropium Bromide	(Ibuprofen)	(Ambroxol)	<(Albuterol, Ipratropium Bromide, Budesonide), (Albuterol, Ipratropium Bromide, Budesonide)>
	**3**	Albuterol	(Albuterol, Ipratropium Bromide, Budesonide, Meptin Syrup)	(Ibuprofen)	<(Albuterol, Ipratropium Bromide, Budesonide), (Ipratropium Bromide, Budesonide)>
	**4**	Ibuprofen	(Albuterol, Budesonide, Meptin Syrup)	(Spasmo-Mucosolvan)	(Albuterol, Ipratropium Bromide, Augmentin, Budesonide)
	**5**	Azithromycin	(Albuterol, Ipratropium Bromide, Meptin Syrup)	(Secodrine Syrup)	<(Ipratropium Bromide, Budesonide), (Azithromycin)>
	**6**	Augmentin	(Albuterol, Ipratropium Bromide, Augmentin, Budesonide)	<(Albuterol, Ipratropium Bromide, Budesonide), (Azithromycin)>	(Ipratropium Bromide, Augmentin, Budesonide)
	**7**	Ceftriaxone	(Ipratropium Bromide, Budesonide, Ambroxol)	(Chloral Hydrate)	<(Ipratropium Bromide, Budesonide), (Albuterol, Ipratropium Bromide, Budesonide)>
	**8**	Cefuroxime	(Cefuroxime, Ipratropium Bromide, Budesonide)	(Pholcodine)	<(Albuterol, Ipratropium Bromide, Budesonide), (Albuterol, Ipratropium Bromide)>
	**9**	Bifid Triple Viable	(Chloral Hydrate)	(Augmentin, Albuterol, Ipratropium Bromide, Budesonide)	<(Albuterol, Ipratropium Bromide, Budesonide), (Albuterol, Budesonide)>
	**10**	Ambroxol	(Pholcodine)	(Smecitite)	<(Albuterol, Ipratropium Bromide, Budesonide), (Ipratropium Bromide)>
**2 to 5 years**	**1**	Azithromycin	(Albuterol, Ipratropium Bromide, Azithromycin, Budesonide)	<(Albuterol, Ipratropium Bromide, Budesonide), (Azithromycin)>	(Albuterol, Ipratropium Bromide, Augmentin, Budesonide)
	**2**	Budesonide	(Ceftriaxone, Ipratropium Bromide, Azithromycin, Budesonide)	(Secodrine Syrup)	(Ipratropium Bromide, Augmentin, Budesonide)

	**3**	Ipratropium Bromide	(Ipratropium Bromide, Azithromycin, Budesonide, Ambroxol)	(Meptin Syrup)	<(Albuterol, Ipratropium Bromide, Budesonide), (Azithromycin), (Azithromycin)>
	**4**	Ibuprofen	(Ipratropium Bromide, Azithromycin, Budesonide, Pholcodine)	(Ibuprofen)	<(Albuterol, Ipratropium Bromide, Budesonide), (Albuterol, Ipratropium Bromide, Budesonide)>
	**5**	Albuterol	(Cefuroxime, Azithromycin)	(Spasmo-Mucosolvan)	<(Albuterol, Ipratropium Bromide, Budesonide), (Ipratropium Bromide, Budesonide)>
	**6**	Ceftriaxone	(Cefuroxime, Ipratropium Bromide, Budesonide)	(Budesonide, Ceftriaxone, Ipratropium Bromide)	(Albuterol, Ipratropium Bromide, Azithromycin, Budesonide)
	**7**	Ambroxol	(Ceftriaxone, Albuterol, Ipratropium Bromide, Budesonide)	<(Ceftriaxone), (Azithromycin)>	(Albuterol, Ipratropium Bromide, Budesonide, Pholcodine)
	**8**	Cefuroxime	(Dexamethasone, Chymotrypsin)	(Augmentin, Ipratropium Bromide, Budesonide)	<(Ipratropium Bromide, Budesonide), (Azithromycin), (Azithromycin)>
	**9**	Augmentin	(Albuterol, Ipratropium Bromide, Budesonide, Pholcodine)	<(Ipratropium Bromide, Budesonide,), (Ipratropium Bromide, Budesonide)>	<(Albuterol, Ipratropium Bromide, Budesonide), (Albuterol, Budesonide)>
	**10**	Pholcodine	(Ipratropium Bromide, Budesonide, Secodrine Syrup)	(Chymotrypsin, Dexamethasone)	<(Albuterol, Ipratropium Bromide, Budesonide), (Albuterol, Ipratropium Bromide)>
**5 years and over**	**1**	Azithromycin	(Ipratropium Bromide, Azithromycin, Budesonide, Ambroxol)	<(Albuterol, Ipratropium Bromide, Budesonide), (Azithromycin)>	<(Albuterol, Ipratropium Bromide, Budesonide), (Azithromycin), (Azithromycin)>
	**2**	Ibuprofen	(Ibuprofen, Ipratropium Bromide, Azithromycin, Budesonide)	(Secodrine Syrup)	<(Ipratropium Bromide, Budesonide), (Azithromycin), (Azithromycin)>
	**3**	Budesonide	(Ibuprofen, Ipratropium Bromide, Azithromycin)	(Meptin Syrup)	<(Albuterol, Budesonide), (Azithromycin), (Azithromycin)>
	**4**	Ipratropium Bromide	(Ceftriaxone, Azithromycin)	(Ibuprofen)	<(Albuterol, Ipratropium Bromide), (Azithromycin), (Azithromycin)>
	**5**	Ceftriaxone	(Dexamethasone, Chymotrypsin)	(Spasmo-Mucosolvan)	<(Albuterol, Ipratropium Bromide, Budesonide, Pholcodine), (Azithromycin)>
	**6**	Albuterol	(Ipratropium Bromide, Augmentin, Azithromycin, Budesonide)	(Budesonide, Ceftriaxone, Ipratropium Bromide)	<(Albuterol, Ipratropium Bromide, Augmentin, Budesonide), (Azithromycin)>

	**7**	Ambroxol	(Albuterol, Ipratropium Bromide, Azithromycin, Budesonide, Pholcodine)	<(Ceftriaxone), (Azithromycin)>	<(Ipratropium Bromide, Budesonide), (Ambroxol)>
	**8**	Pholcodine	(Ceftriaxone, Albuterol, Ipratropium Bromide, Azithromycin, Budesonide)	(Augmentin, Budesonide, Ipratropium Bromide)	<(Ipratropium Bromide, Augmentin, Budesonide), (Azithromycin)>
	**9**	Augmentin	(Azithromycin, Spasmo-Mucosolvan)	<(Ipratropium Bromide, Budesonide), (Ipratropium Bromide, Budesonide)>	<(Albuterol, Ipratropium Bromide, Budesonide), (Pholcodine)>
	**10**	Cefuroxime	(Azithromycin, Chymotrypsin)	(Chymotrypsin, Dexamethasone)	<(Albuterol, Ipratropium Bromide, Budesonide), (Ibuprofen), (Ibuprofen), (Azithromycin)>
